# Supplementary material for: Analysis of lifespan across diversity outbred mouse studies identifies multiple longevity-associated loci
Source: Genetics. 2025 May 6;230(4):iyaf081. doi: 10.1093/genetics/iyaf081 (PMC12342377; doi:10.1093/genetics/iyaf081)
Supplement: iyaf081_Supplementary_Data [file iyaf081_supplementary_data.zip › Supplemental_Material_Legends_GENETICS-2025-307990.docx]

**SUPPLEMENTARY FIGURE LEGENDS**

**Figure S1 | False discovery rate (FDR) across LOD scores in each of the individual lifespan studies and mega-analysis.** Ribbons around each line correspond to the standard error of the estimate.

**Figure S2 | Effects of peak markers at additional QTLi (DRiDO study) on lifespan.** Effects (BLUPs) and corresponding standard errors for animals containing at least one copy of the corresponding founder allele.

**Figure S3 | Association mapping of nominally significant QTL from a mega-analysis of lifespan. a,** Variant association mapping of the QTL on chromosome 16.2 depicting the LOD scores for each variant within the 3 LOD support interval around the locus in the mega-analysis (*top*). The area encompassed by the plot corresponds to the 3 LOD support interval around the locus in the mega-analysis. The most likely candidate SNPs are highlighted in pink. Genes within the genomic interval are depicted (*bottom*). **b,** Variant association mapping of the chromosome 2 locus in the mega-analysis. **c,** Variant association mapping of the chromosome 4 locus in the mega-analysis.

**Figure S4 | QQ plot of -log_10_(p) values from the GxDiet mixed effects model plotted against -log_10_(p) values sampled from a uniform distribution.** The red line indicates the nominal threshold chosen to determine significance.

**SUPPLEMENTARY DATA / TABLES**

*Data S1:*

Lifespan and covariate data for the mice in each of the four studies. ‘Study’ corresponds to the study the animal was enrolled in: ‘Shock’, ‘Harrison’, ‘Svenson’, or ‘DRiDO’ (Dietary Restriction). ‘Mouse.ID’ corresponds to the unique identifier assigned to each animal. ‘Sex’, ‘Diet’, and ‘Generation’ are the primary covariates used in the analysis; ‘Generation’ corresponds to the specific cohort or generation wave of the DO mice. ‘Status’ reflects the outcome of animals as they exit their respective study; ‘1’ means the animal died and ‘0’ means the animal was censored.

*Data S2:*

Eight-state allele probabilities from the Harrison study.

*Data S3:*

Lifespan and covariate data for the Harrison study.

*Data S4:*

Eight-state allele probabilities from the Shock study.

*Data S5:*

Lifespan and covariate data for the Shock study.

*Data S6:*

Eight-state allele probabilities from the Dietary Restriction (‘DRiDO’) study.

*Data S7:*

Lifespan and covariate data for the Dietary Restriction (‘DRiDO’) study.

*Data S8:*

Combined eight-state allele probabilities from the Harrison, Shock, and DRiDO studies.

*Data S9:*

Combined lifespan and covariate data for the Harrison, Shock, and DRiDO studies.

*Table S1:*

Test statistics for log rank tests comparing lifespan among female mice fed an *ad libitum* diet by study. ‘Test’ refers to the pair of studies being tested. ‘chi_sq’ and ‘pval’ columns provide the ꭓ^2^ and p-value associated with each test.

*Table S2:*

Heritability estimates and standard errors for individual treatment groups in the Harrison, Shock, and DRiDO study. ‘Group’ corresponds to the individual treatment group, and follows the naming convention of Study_Sex_Intervention, where intervention refers to diet or rapamycin treatment. ‘h2’ corresponds to the heritability estimate within the treatment group. ‘h2_se’ corresponds to the standard error of the heritability estimate.

*Table S3:*

List of QTL detected in the Harrison, Shock, and DRiDO studies, the mega analysis, and the GxEMM scans. ‘Study’ refers to the particular dataset in which a QTL was detected. ‘chr’ refers to the chromosome on which the QTL is located. ‘pos’ refers to the physical location on the respective chromosome at which the QTL is located in megabases (Mb). ‘lod’ refers to the LOD score of the peak marker at the QTL. ‘ci_lo’ refers to the location of the marker denoting the 2LOD drop prior to the peak marker. ‘ci_hi’ refers to the location of the marker denoting the 2LOD after the peak marker. ‘threshold’ refers to the significance threshold used to call the peak marker of the QTL, which can be ‘Nominal’, ‘Permutation’, or ‘GxEMM’. ‘Nominal’ corresponds to a conservative threshold of p<= 1*10^-6^, ‘Permutation’ corresponds to an alpha critical threshold of <=0.05 based on 1,000 permutations of the data, and ‘GxEMM’ corresponds to the previously reported threshold of p <= 1*10^-4^.

*Table S4:*

Table of genes underlying each QTL. ‘study’ corresponds to the study in which a particular QTL was mapped. ‘qtl_id’ corresponds to the chromosome and position at which the QTL was mapped, separated by a “_” character. ‘chr’ corresponds to the chromosome on which the QTL was mapped. ‘source’ corresponds to the database from which gene annotation data was collected. ‘type’ corresponds to the annotation associated with a marker (can be ‘gene’ or ‘pseudogene’). ‘start’ and ‘stop’ correspond to the first and last physical coordinates associated with the annotation in megabases (Mb), respectively. ‘strand’ corresponds to which strand of DNA the annotation is on. ‘ID’ corresponds to the database ID assigned to the annotation. ‘Name’ corresponds to the gene name associated with the annotation. ‘Dbxref’ lists annotation IDs in other databases. ‘gene_id’ corresponds to the gene ID in the corresponding database. ‘mgi_type’ corresponds to the gene type associated with the annotation in the corresponding database. ‘description’ provides a functional description of the protein or RNA associated with the annotation.

*Table S5:*

Statistical test for interaction between chromosome 16 and sex in the Shock study.
