# Supplementary figures and images for: Analysis of lifespan across diversity outbred mouse studies identifies multiple longevity-associated loci
Source: Genetics. 2025 May 6;230(4):iyaf081. doi: 10.1093/genetics/iyaf081 (PMC12342377; doi:10.1093/genetics/iyaf081)

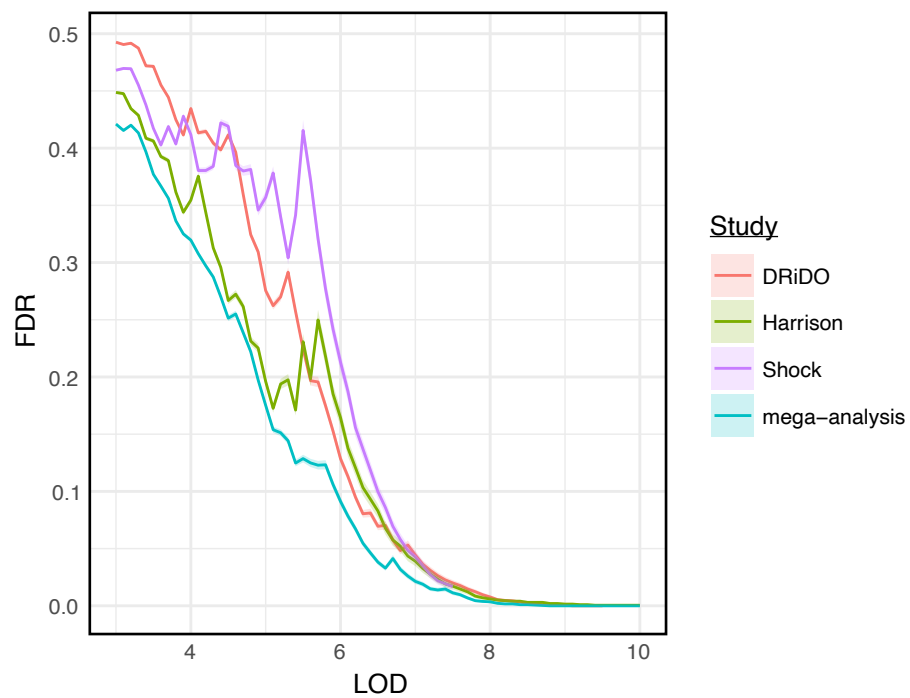

Supplement: iyaf081_Supplementary_Data [file iyaf081_supplementary_data.zip › Figure_S1_GENETICS-2025-307990.pdf]

**Chromosome 7 – pos: 11385192**

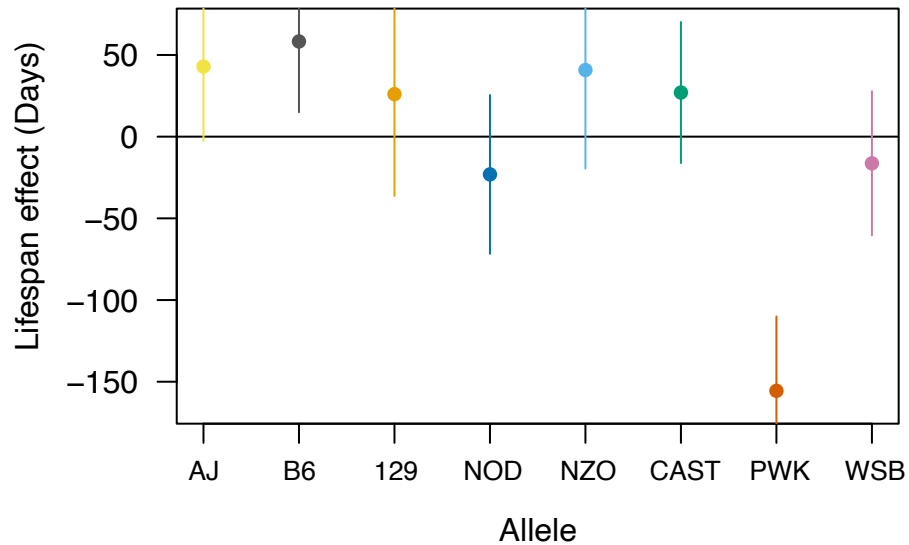

**Chromosome 7 – pos: 108568535**

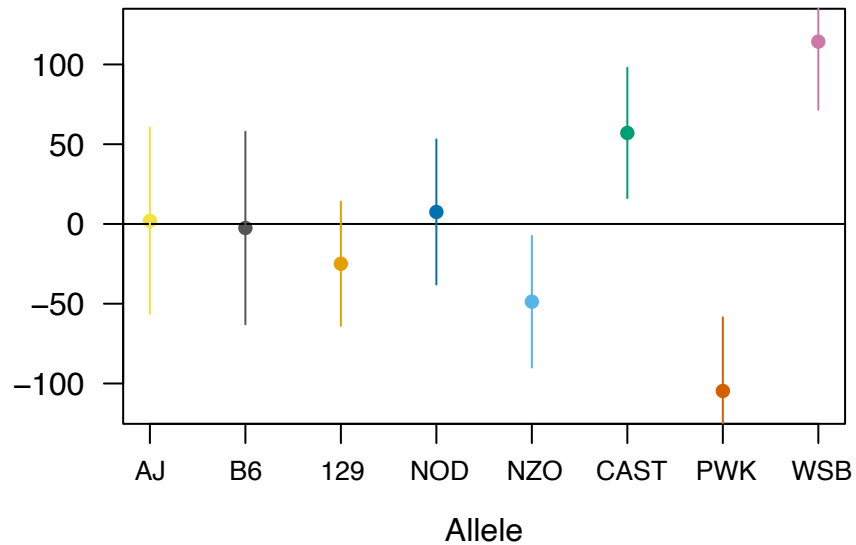

Supplement: iyaf081_Supplementary_Data [file iyaf081_supplementary_data.zip › Figure_S2_GENETICS-2025-307990.pdf]

# LOD Score

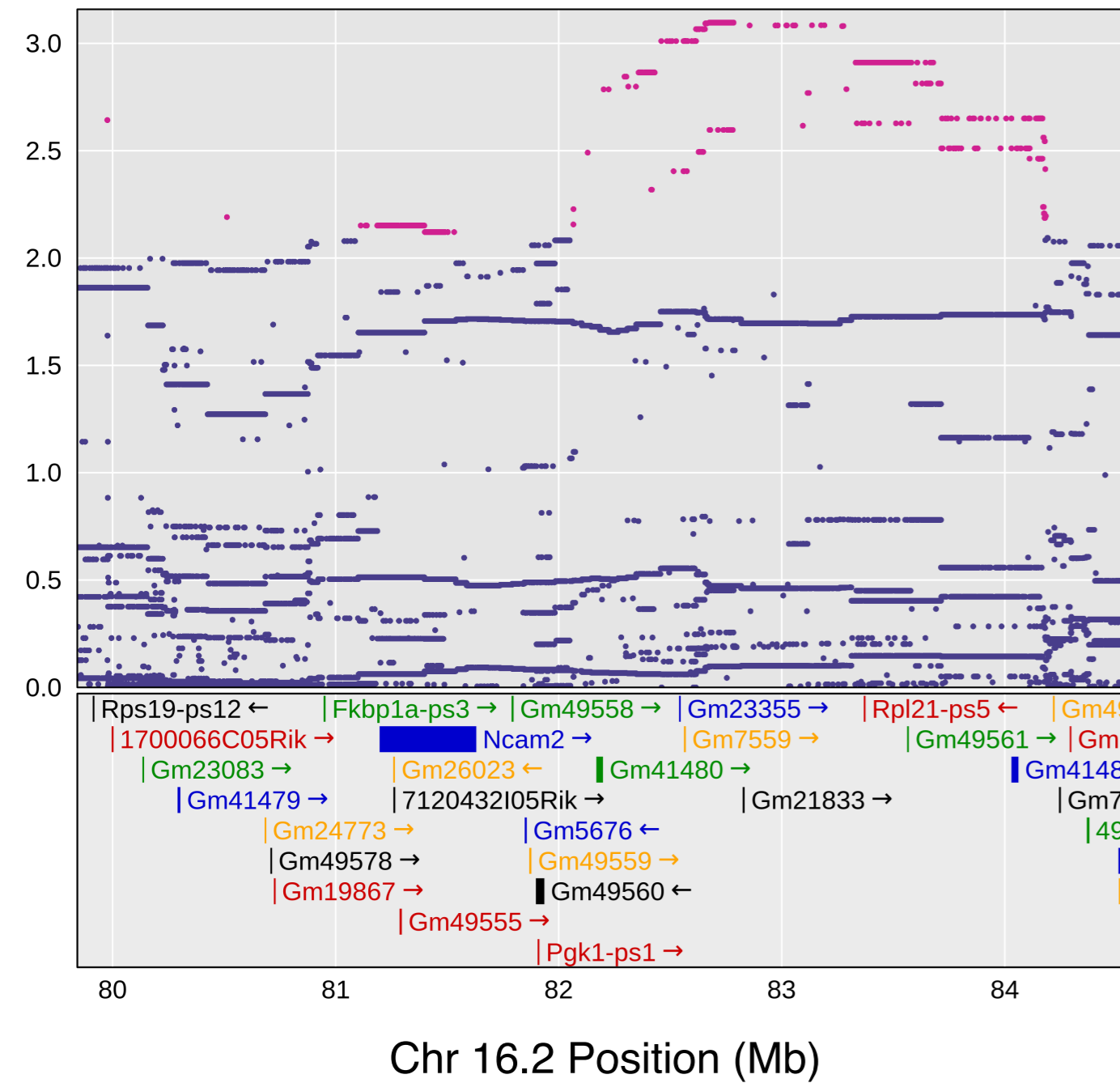**b**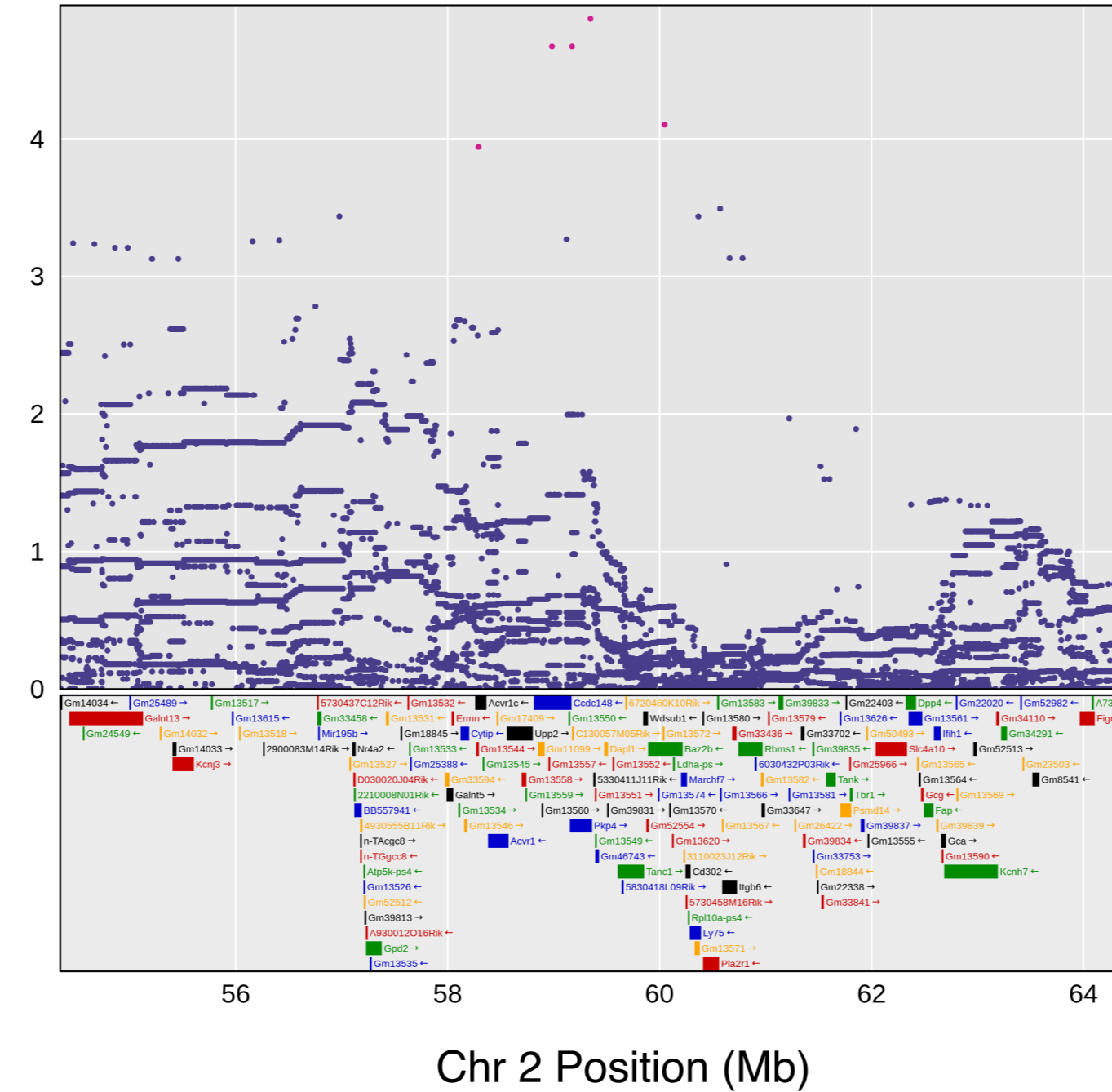

**C**

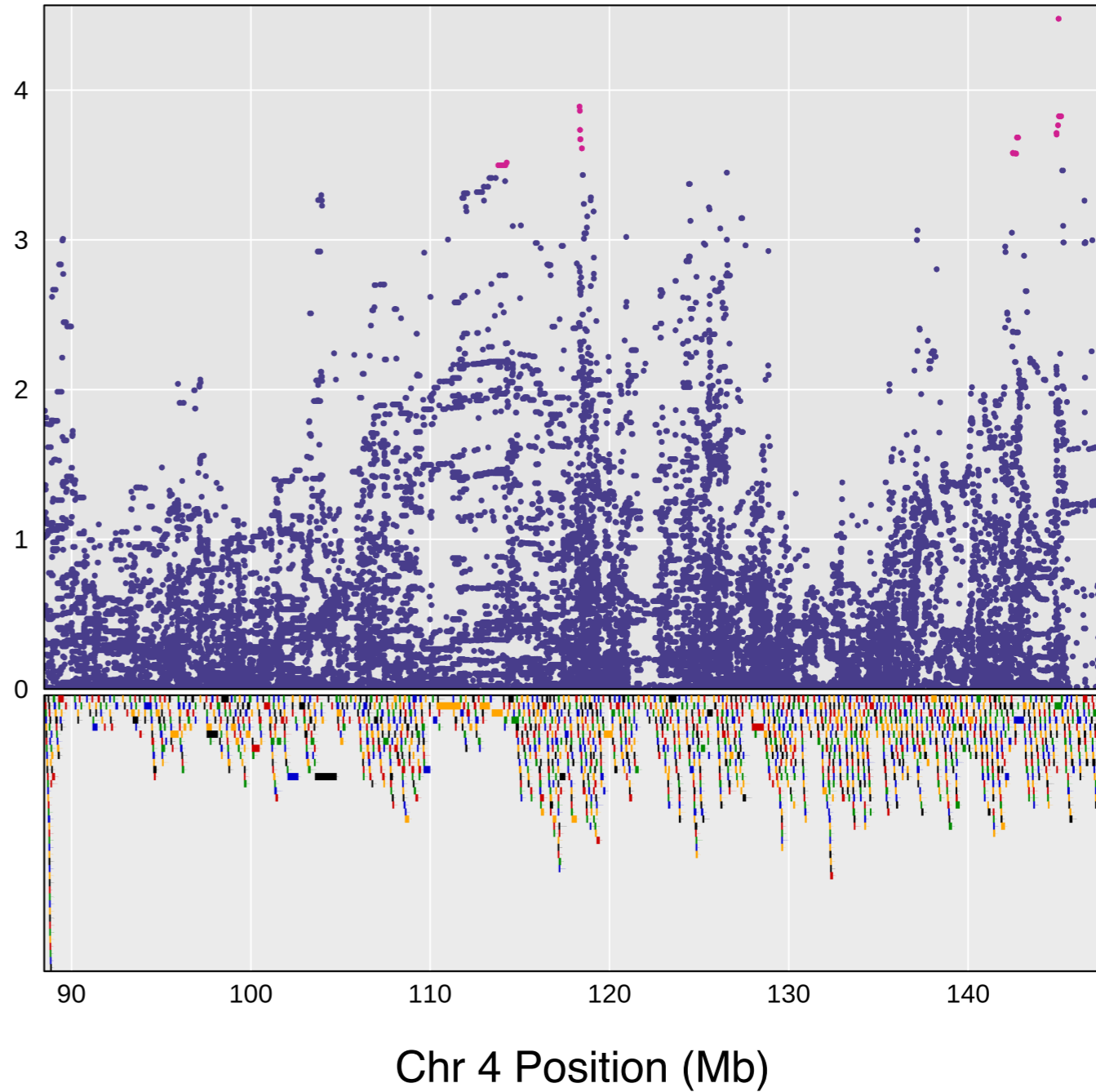

Supplement: iyaf081_Supplementary_Data [file iyaf081_supplementary_data.zip › Figure_S3_GENETICS-2025-307990.pdf]

GxEMM - G x Diet Model

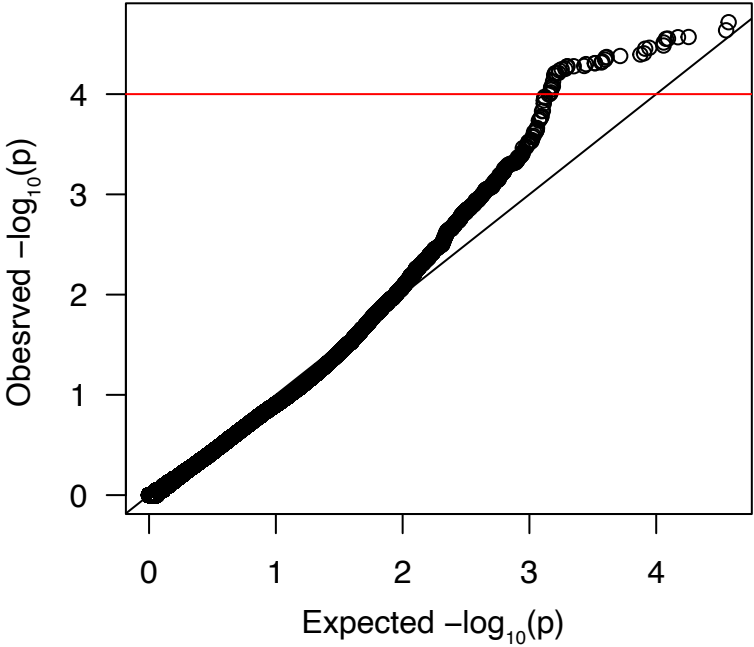

Supplement: iyaf081_Supplementary_Data [file iyaf081_supplementary_data.zip › Figure_S4_GENETICS-2025-307990.pdf]
